# Chest Imaging in the Diagnosis and Management of Pulmonary Tuberculosis: The Complementary Role of Thoraci Ultrasound

**DOI:** 10.3389/fmed.2021.753821

**Published:** 2021-12-10

**Authors:** Gaetano Rea, Marco Sperandeo, Roberta Lieto, Marialuisa Bocchino, Carla Maria Irene Quarato, Beatrice Feragalli, Tullio Valente, Giulia Scioscia, Ernesto Giuffreda, Maria Pia Foschino Barbaro, Donato Lacedonia

**Affiliations:** ^1^Department of Radiology, Azienda Ospedaliera dei Colli-Cotugno and Monaldi Hospital, Naples, Italy; ^2^Department of Medical Sciences, Unit of Interventional and Diagnostic Ultrasound of Internal Medicine, Istituto di Ricovero e Cura a Carattere Scientifico (IRCCS) Fondazione Casa Sollievo della Sofferenza, San Giovanni Rotondo, Foggia, Italy; ^3^Respiratory Medicine Unit, Department of Clinical Medicine and Surgery, Federico II University, Naples, Italy; ^4^Department of Medical and Surgical Sciences, Institute of Respiratory Diseases, Policlinico Universitario “Riuniti” di Foggia, University of Foggia, Foggia, Italy; ^5^Department of Medical, Oral and Biotechnological Sciences - Radiology Unit “G. D'Annunzio”, University of Chieti-Pescara, Chieti, Italy

**Keywords:** pulmonary tuberculosis, diagnosis and management, chest imaging, chest X-ray (CXR), chest computed tomography (chest CT), thoracic ultrasound (TUS)

## Abstract

Tuberculosis (TB) is a severe infectious disease that still represents a major cause of mortality and morbidity worldwide. For these reasons, clinicians and radiologists should use all the available diagnostic tools in the assessment of the disease in order to provide precise indications about starting an anti-tubercular treatment and reduce risk of TB transmission and complications especially in developing countries where the disease is still endemic. As TB mycobacteria are mainly transmitted through respiratory droplets, the pulmonary parenchyma is usually the first site of infection. As a result, chest imaging plays a central role in the diagnostic process. Thoracic ultrasound (TUS) is a portable, non-invasive, radiation-free, and cost-contained technology which could be easily available in resource-limited settings. This perspective article focuses on the potential role of TUS in the diagnosis and management of patients with pulmonary TB. Unfortunately, there are still insufficient evidence and too contrasting data to judge TUS as an appropriate diagnostic method for the screening of the disease. Despite this, TUS may have a useful role in identifying pleural and anterior pericardial effusions or in the identification of abscesses of the anterior chest wall and paraspinal collections in low- and middle-income settings. In addition, TUS seems to have a milestone role in guiding minimally invasive interventional procedures, such as placement of chest tubes, drainage of loculated collections, thoracentesis and pericardiocentesis, and percutaneous biopsy of subpleural pulmonary consolidations or pleural plaques.

## Introduction

Tuberculosis (TB) is a severe infective disease that still represents a “world emergency” despite large investments in care over the past three decades. According to the latest report by the World Health Organization (WHO), it is estimated that in 2019, there were 130 new cases of TB per 100,000 population globally. Most of the new cases are found in Asia and Africa (1). Without appropriate treatment, the mortality rate from TB is high. Moreover, an untreated TB implies, above all, several complications and sequelae that, in the short or long term, are capable of leading to functional and morphological, focal, or diffuse alterations, severely impacting patients' outcome. These include lung post-infectious fibrosis, fibrothorax, bronchiectasis, cavitations, calcific granulomas, dense fibrotic bands, and areas of air-trapping due to post-infectious obstructive bronchiolitis, broncho-pleural fistulas, and lung cancer (scar cancer) (2).

Causative agents of TB are acid-fast aerobic bacilli belonging to the Mycobacterium Tuberculosis Complex (MTC), mainly including *M. tuberculosis* [and, to a lesser extent, *M. africanum* (3)]. The main gateway of infection in humans is the respiratory tract, by inhalation of aerosolized droplets containing mycobacterium bacilli. These droplets, coming from secretions of the respiratory tract of infected subjects, may remain suspended in the air for a long time, and, given their size, are able to reach small airways and the alveolar compartment (4).

The clinical course and the eventual different manifestations of TB infection mainly depend on immune status of the individual, genetic, and charge of exposure to the microorganism. On average, 5–10% of infected subjects will develop active TB disease over the course of their lives, usually within the first 2–5 years after initial infection (5, 6). The biggest risk factors for TB reactivation include advanced age, malnutrition, cancer, impaired immunity, AIDS, diabetes, and immunosuppressive treatments, including anti-TNF-α therapies (7, 8). A tuberculin skin test (TST) or interferon-gamma release assay (IGRA) can be used for the screening of latent TB. However, these tests can lead to false-negative results, particularly in young children and immune-compromised individuals. “Active” tuberculosis is diagnosed by isolating mycobacterium bacilli from bodily secretions.

Although TB is capable of affecting any organ in the human body, the first site of TB mycobacteria infection is usually the pulmonary parenchyma. As a consequence, chest imaging plays an important first line role for an early diagnosis of the disease. Chest X-ray remains the first instance method for detecting a suspected tuberculosis, although the exam may be normal or show only mild or non-specific findings in patients with active disease. Chest CT, with or without contrast enhancement, may be helpful for better characterization of radiographic findings by helping to distinguish between previous inactive and active disease or in detecting alterations that cannot be assessed on standard radiograph. “Active” disease is generally characterized by the presence of centrilobular nodules, tree-in-bud pattern, thick-walled cavities, consolidations, miliary nodules, pleural effusions, or necrotic lymphadenopathy (9). “Inactive” tuberculosis is characterized by 6-month stable alterations, including scarring (i.e., peri-bronchial fibrosis, bronchiectasis, and architectural distortion) and nodular opacities (i.e., calcified granulomas and calcified lymph nodes) (10, 11). Anyhow, as clinical and radiological signs of TB may mimic those of many other diseases (i.e., lymphoma and other neoplasms or granulomatous diseases and such as sarcoidosis), reaching a specific diagnosis may be challenging (12). Furthermore, in patients with latent TB infection it is not uncommon to detect fibronodular changes especially in the lung apices, making the distinction between active from inactive disease very difficult.

In the last years, thoracic ultrasound (TUS) has gained great interest from both clinicians and radiologists as a useful diagnostic tool for the study of many pleuro-pulmonary conditions (13). Although TUS examination has not yet a standardized role in the diagnosis and management of pulmonary TB in international guidelines, this imaging technology is advantageous in terms of non-invasiveness and cost-containment, is suitable for a quick real-time evaluation, and is a portable technology readily available for all clinicians in all hospital wards. This makes TUS a potentially useful diagnostic tool for the diagnosis of pulmonary TB, particularly in geographic areas where the access to radiological or laboratory tests is limited. Moreover, TUS has the advantages of not exposing children and pregnant women to ionizing radiation, also representing a diagnostic method that allows considerable improvement in safety for patients who need frequent follow-ups.

The aim of this perspective article is to discuss about the potential role of TUS in the field of tuberculosis diagnosis and management.

## Imaging Findings

Imaging findings of chest TB have been classified in lymphadenopathy, parenchymal disease, pleural effusion, pericardial effusion, miliary TB, abscesses of the chest wall, and paraspinal collections. Existing literature and main field of TUS application for each finding have been analyzed and discussed.

## Lymphadenopathy

Lymph node enlargement is seen in up to 96% of children and 43% of adults, being typically unilateral and right-sided (14). The most affected lymph node stations are the hilum and right paratracheal region. Although lymphadenopathy is usually associated with other manifestations of TB, it may be the sole radiographic feature. This occurs more common in children (15, 16).

Given the advantage of no exposure to ionizing radiation, TUS have been suggested for the assessment of mediastinal lymphadenopathy in children. In 2004, Bosch-Marcet et al. (17) reported success in evaluation of enlarged lymph nodes (with round or oval hypoechoic appearance) in the paratracheal region and aortopulmonary window, because of a good acoustic view. However, ultrasound was not a good technique for assessing hilar involvement, because the hila are surrounded by air in the lungs and only large lymph nodes were accessible in the subcarinal region due limitations in the sonographic access (e.g., the esophagus). Despite these limitations, the reported diagnostic performance of TUS in assessing mediastinal lymphoadenopathy was 90.5%. Later, the same group of authors proposed TUS also as an effective non-invasive method for the control and follow-up of mediastinal lymph nodes of children receiving antituberculous chemotherapy (18). More recent studies showed contrasting result, with sensitivity values ranging from 19 to 40% (19, 20). Despite mediastinal lymph nodes enlargement can also be seen in children with other lower respiratory tract infection, the study of Heuvelings et al. (19) highlighted a moderate specificity (71.9%) for this TUS finding.

If the data relating to the use of TUS are contrasting and do not allow to draw clear conclusions, endobronchial ultrasound-guided transbronchial needle aspiration (EBUS-TBNA) is instead an established minimally invasive method for the assessment of mediastinal/hilar lymphadenopathy in adult with TB. The diagnostic accuracy of the procedure ranges from 68 to 94% among the various studies (21–27). More limited literature is available on the utility of EBUS-TBNA in children, with a diagnostic accuracy ranging from 36 to 100% (28–32). Some authors reported that the finding of a heterogeneous echotexture at EBUS or the identification of coagulation necrosis on the granulomatous biopsy could help in the differential diagnosis of tuberculosis compared to other lymph node pathologies, such as sarcoidosis or lymphoma (33, 34). The addition of TB cultures and a nucleic acid amplification (NAA) test, such as the Xpert MTB/RIF assay, can increase the specificity for the diagnosis of mediastinal tuberculous lymphadenopathy and its drug-resistant form (35).

## Parenchymal Disease

In studies examining adult patients with pulmonary TB, the most frequent ultrasound parenchymal findings were subpleural nodules and lung consolidations, while the detection rate of cavitations was low (36–40). A recent systematic review calculated for TUS a sensitivity ranging from 72.5 to 100.0% in the assessment of subpleural nodules and from 46.7 to 80.4% in the detection of consolidations. On the other hand, TUS sensitivity for cavitations ranged from 4.0 to 30.0% (41).

Only the study from Heuvelings et al. (19) provided detailed data on TUS findings in pediatric pulmonary TB. Consolidations were the most common parenchymal alteration in children with pulmonary TB and TUS showed a sensitivity of 45.6% in their detection.

Lung consolidations are visible on ultrasound only if they are faced to the superficial pleura. In this context, TUS may be used in assessing the response to therapy during the follow-up of the patients examined, thus configuring a suitable and repeatable alternative to further radiation exposure, that is useful specially in children and pregnant women (19, 42–44). However, lung lesions that are not adjacent to the pleura cannot be imaged due to the subtotal reflection that intrapulmonary air determines on the pathway of the ultrasound beam (45, 46). TUS sensitivity for the detection of cavitations appears low in all the studies probably due to the high number of lesions not reaching the pleura. Moreover, the differential diagnosis between the consolidations can be really wide due to the similarities of ultrasound appearance between benign, borderline, or malignant lesions, and TUS specificity in the distinction of consolidation changes (i.e., supervening processes of necrosis, fibrosis, and calcification) is low (42, 45, 46). According to the study from Montuori et al. (39), in adult with pulmonary TB, the specificity of subpleural nodules, lung consolidations, and cavitations were 66.7, 25.3, and 89.3%, respectively. According to Heuvelings et al. (19) the specificity of lung consolidations in children was 53.3%.

Bronchogenic spread may be a complication. In such cases, chest CT allows the identification of multiple micronodules with a centrilobular distribution associated to sharply margined linear branching opacities in the context of the small airways (“tree-in-bud” sign). The histopathological correlate of centrilobular nodules is the presence of granulomatous inflammation and caseous necrosis within and around terminal and respiratory bronchioles, while peripheral linear branching opacities correspond to tuberculous bronchitis of the small airways (10). These findings are considered indicative of active tuberculosis (47). The “tree-in-bud” pattern usually involves lower lung lobes as centrilobular nodules are peripheral, but spare the subpleural lung (47). Clearly, a diagnosis of small airway disease cannot be carried out with TUS that, in this context, may show only the presence of irregular signs of interface, such as pinching and irregularities of the pleural line and non-specific artifacts including comet tails and ring down (B-lines) (48).

Although common TUS findings of pulmonary TB are not enough specific, this imaging modality can still be helpful in US-guided transthoracic cutting biopsy for histological and microbiological characterization (45, 49, 50). In the literature, biopsies of TB lesions resulted in moderate to high rates of success, ranging from 77 to 90% (36, 51).

## Pleural Effusion

Pleural effusion (usually resulting from hypersensitivity to mycobacterium antigens) is typically unilateral (14, 16). On Chest X-ray, this finding is usually associated with parenchymal disease. Pleural effusions can remain stable in size for many years, develop septations due to fibrin strands, and result in residual pleural thickening and calcification (14).

Thoracic ultrasound (TUS) is a high effective way in detecting pleural effusion even of modest entity, resulting ultrasound to perform better than chest X-ray. Furthermore, TUS is a fast and useful method not only in differentiating effusion from consolidated lung (whereas both appear “white” on chest radiographs), but also in evaluating the “quality” of liquid alterations. Pleural effusion on ultrasound can appear as anechoic (black), complex non-septated (black with white strands), complex septated (black with white septa), or homogeneously echogenic (white) (52). In general, the presence of homogeneous echogenic effusion suggests corpuscular effusion (i.e., hemorrhage or empyema), whereas an anechogenic effusion might be transudative. Since 1989, several studies demonstrated that TUS can be useful in detecting sepimentations, loculations, and thickenings of the pleura in adult patients with pulmonary TB (53–56). However, these studies did not provide data on TUS sensitivity compared to radiographic “gold standard” methods.

**Figure 1:**
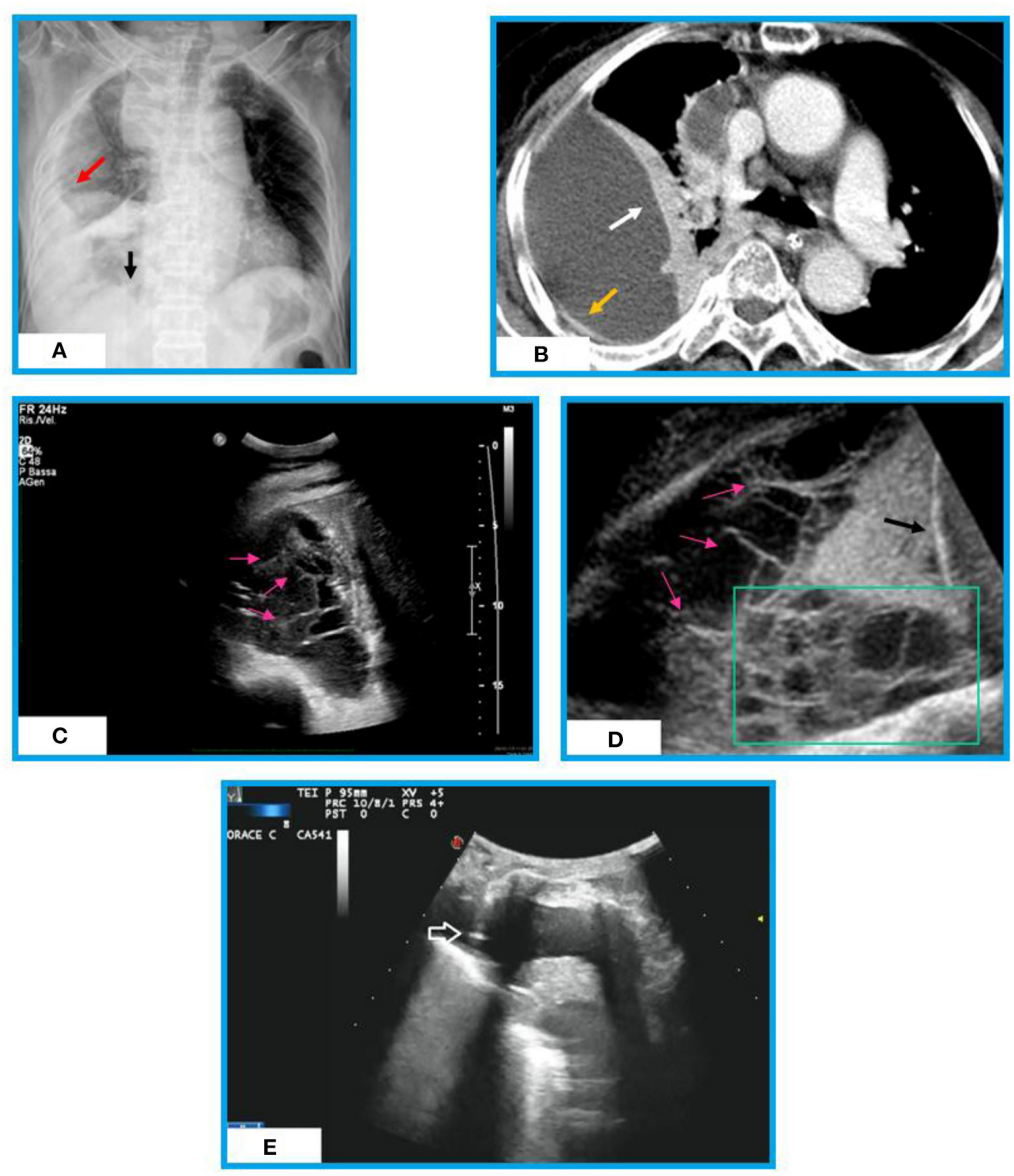
Chest X-ray, chest CT and Ultrasound appearances of an organized effusion in a patient with post-primary TB. **(A)** Chest X-ray shows a right pulmonary opacity that is not in the gravity dependent location (red arrow). We are able to see the diaphragm medially (black arrow). No septation is seen. **(B)** Chest CT shows a large loculated right pleural effusion surrounded by a thickened pleural wall (yellow arrow) and lung atelectasis (white arrow). No septation is assessed inside. **(C)** Thoracic ultrasound (TUS) scan reveals presence of multiple fibrin strands (i.e., thin, mobile, linear hyperechoic structures, pink arrows) forming a septated pleural effusion (complex US aspect). **(D)** TUS lower thoracic view (diaphragm, black arrow) of the pleural effusion showing multiple septations (pink arrows) and loculations (green box). **(E)** TUS scan during ultrasound-guided thoracentesis allowing real-time visualization of the needle (white arrow) in an anechoic pleural effusion.

According to a recent study from Zhou et al. (57), B-mode ultrasound was less effective than Chest CT in assessing the thickening of the parietal pleura, but there were no substantial differences for pleural effusions and pleural calcifications. The sensitivity values of TUS in detecting pleural effusions, pleural calcifications, and pleural thickenings compared to Chest CT were 77.69, 74.01, and 2.77%. The sensitivity and specificity of the combined three signs in assessing TB pleurisy was 85.51 and 72.34%, respectively. Moreover, although specific data in TB patients are not yet available in the current literature, ultrasound showed a higher sensitivity than chest X-ray and CT scan in detecting fibrin strands and multi-loculation that develop in protein-rich exudative effusions (58, 59) (Figures 1A–D).

In addition, TUS is a particularly useful real-time method in guiding thoracentesis needle for risk-free sampling of liquid on which cytologic examination (i.e., lymphocytic effusions are suggestive) and chemical analysis [i.e., determination of pleural fluid adenosine deaminase (ADA) level] can be performed (60, 61) (Figure 1E). Chen et al. (62) found that a complex septate pattern at TUS is a useful predictor of TB in lymphocyte-rich exudative pleural effusions. A chest tube for evacuative thoracentesis can be safely inserted under TUS guide for the drainage of loculated collections (45, 52).

If the results of fluid analysis are not definitive, TUS may be used for transthoracic sample of pleural specimens that can be examined for the histological assessment of granulomas and can be cultured for organisms (63, 64). Ultrasound-assisted Abrams' needle biopsy still seems to be the better choice in allowing the diagnosis of pleural tuberculosis compared to ultrasound-guided Tru-Cut needle biopsy (65–67). Sun et al. (68) showed that the Xpert MTB/RIF assay may increase the diagnostic sensitivity for TB in histological pleura samples obtained through contrast enhanced ultrasound (CEUS) guided biopsy. CEUS, on turn, demonstrated to be an efficient, minimally invasive, and safe method for guide biopsy.

## Pericardial Effusion

Tuberculosis (TB) is an important cause of pericardial effusions, especially in HIV-infected patients and children. Therefore, an extensive TUS examination, including also a cardiac evaluation, may be an useful cost-effective and radiation-free method to diagnose pericardial effusions in patients with HIV-associated TB and children, especially in geographic areas where TB is endemic (69–73). Ultrasonographically, pericardial effusion appears as an anechoic black stripe around the heart separating the visceral and parietal pericardium. Although Zhou et al. (57) reported that CT examination can more accurately show pericardial effusion in patients with tuberculous pleuritis compared with B-mode ultrasound, pericardial ultrasound examinations showed to be a valuable supplementary investigations in the diagnosis of suspected extrapulmonary or disseminated TB in South Africa (74–76). Ultrasound may also be employed as a safe and cost-effective guide for pericardiocentesis in low and middle income settings (77, 78). Furthermore, ultrasound follow-up may be very useful for monitoring treatment response in children with extrapulmonary TB (73).

## Miliary TB

**Figure 2:**
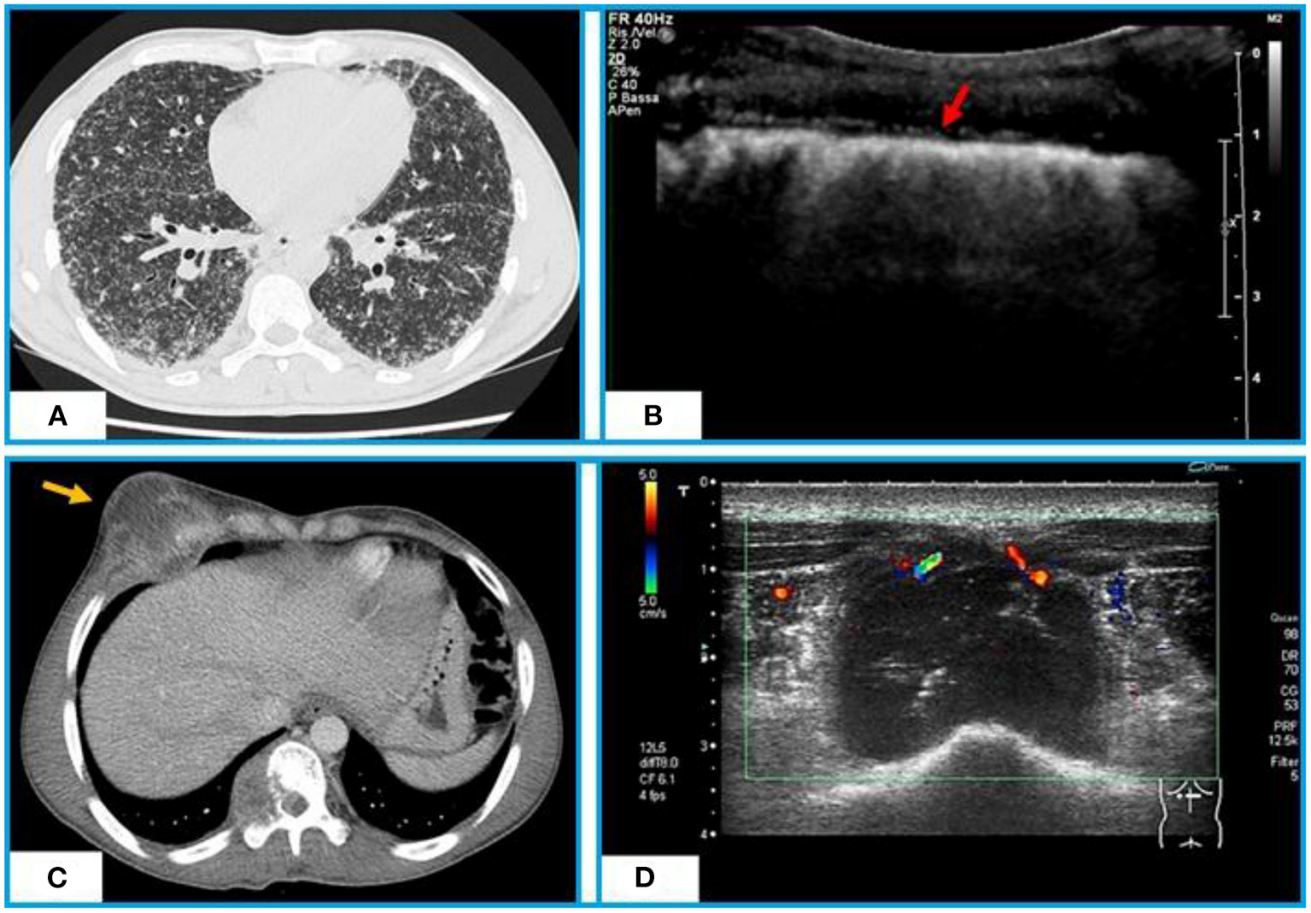
Chest CT and TUS appearance of miliary TB. **(A)** Axial CT scan shows hundreds of minutes “millet grain” nodules with a typical random distribution. **(B)** TUS highlights a marked irregularity of the hyperechoic pleural line (red arrow), that appears as blurred and fragmented. Chest CT and TUS appearance of a tuberculous abscess of the anterior chest wall. **(C)** Axial CT scan with contrast shows a tuberculous abscess with a low-attenuation central necrotic component and capsular ring enhancement extending from the pleura to right chest wall and infiltrating the rib and soft tissues. **(D)** TUS scan shows a hypoechoic collection in right antero-lateral chest wall with internal echo-debris and no Color-Power Doppler inner signal.

Miliary pulmonary TB is a severe form of disease that occurs in ~1–7% of TB. It mainly affects the elderly, immunocompromised hosts, and infants. The typical feature of this disease, on both chest X-ray and chest HRCT, is the presence of hundreds of minutes “millet grain” nodules without a preference for anatomical lung location and distributed in both lungs from the apexes to the bases. On High Resolution Chest Tomography (HRCT), many of these nodules are close to fissures and pleural layers with a typical random distribution (14). This pattern is caused by the massive hematogenous dissemination of TB. TUS may highlight slight or marked irregularity of the hyperechoic pleural line that appears as blurred, fragmented, and thickened from the apex to the base in each lung. Moreover, tiny subpleural nodules in miliary TB can determine a non-specific ultrasound pattern characterized by an increased number of discrete B-lines or a blurred or irregular coalescence of multiple artifacts (i.e., B-lines, comet tails, A-lines in the same scan image) (37, 38, 40, 79). However, these ultrasound aspects do not allow to differentiate military TB from other HRCT interstitial lung patterns [i.e., Usual Interstitial Pneumonia (UIP), Non-Specific Interstitial Pneumonia (NSIP), Chronic Hypersensitivity Pneumonia (CHP) or others] (80). The marked irregularity of the pleural line, with a thickened, fragmented, and/or blurred appearance, may suggest also an aggressive or advanced stage of fibrotic disease (81). As a result, TUS examination alone may be dangerous and confounding. Only clinical evaluation, laboratory data, and more confidential radiological assessment (Chest X-ray and HRCT) with a correct multidisciplinary approach can allow a correct diagnosis (Figures 2A,B).

## TB Abscesses

Due to the systemic nature of tuberculosis, the infection can involve numerous parts of the body, including ribs, sternum, and soft tissue of the chest wall. Typical presentations include abscesses and chronic sinus formation, which are often secondary to lung, pleura, or mediastinal TB. Similarly, iliopsoas abscess is a common complication of Pott's spine (vertebral tuberculosis) in developing countries where the tuberculosis is still an endemic disease (82). TUS, with B-mode examination, may image TB abscesses as hypoechoic areas, with varying degrees of internal heterogeneity (83). Color-Power Doppler well highlights the possible presence of vascular signals (Figures 2C,D). TUS guided percutaneous drainage represents a safe and radiation-free modality of treatment for abscesses of the anterior chest wall and paraspinal collections, allowing also to clarify their nature (84, 85).

## Discussion

For a screening method suitable for pulmonary TB, the WHO recommends a minimum of 90% sensitivity and 70% specificity (86). The analysis of the available literature shows that there is still insufficient evidence to judge the diagnostic accuracy of TUS for a screening purpose in TB. The sensitivity of TUS findings between the studies is too varied. This variability may be due to different setting of ultrasound scanners and variable protocols of image acquisition or may imply operator dependence. Furthermore, under optimal conditions, ultrasounds are able to highlight only the 70% of the pleural surface and everything that closely faces this surface (45, 87). Some authors seem to display excessive confidence in the ultrasound examination. As a first step in proper TUS scanning, it is crucial that the operator is accurately trained and has a good understanding of ultrasound physics in order to be able to correctly set-up the ultrasound scanner implementing the diagnostic capabilities of the exam (45, 46). Moreover, the operator should evaluate the patient in terms of physical constitution (i.e., obesity and conformation of the rib cage) and the presence of conditions potentially limiting the examination (i.e., obliged decubitus and presence of dyspnoea) (88). To date, a diagnosis of active TB is accurately highlighted exclusively by clinical context, laboratory and microbiological texts, and standard radiologic exams, including chest X-ray and CT. Despite this, the use of TUS may have a good rationale in particular circumstances that include avoiding exposure to ionizing radiation in children, pregnant women, and patients who need frequent follow-ups and cost-containing in countries with few economic availabilities. Anyhow, the milestone in the employment of TUS in chest TB seems to be represented by its role as a guide in minimally invasive interventional procedures.

Due to the paucity and contradiction of available data, no conclusions can be drawn on diagnostic accuracy of TUS in identifying pathological mediastinal lymph nodes in children with suspected pulmonary TB. In 2017, the group of Pool et al. (89) proposed a protocol for imaging mediastinal lymphadenopathy that was employed only in the study from Heuvelings et al. (19) in children with pulmonary TB. Further studies are needed for validation. Contrary, EBUS-TBNA is a widely accepted technique for sampling mediastinal and hilar lymphadenopathies in adult with TB. More studies on the pediatric population would be desirable in order to confirm the few, but encouraging, currently available data.

Regarding parenchymal ultrasound patterns, subpleural nodules were also described in other acute infective or chronic pulmonary diseases, and the ultrasound pattern does not allow to differentiate the nature of the consolidations. Moreover, ultrasound is clearly unable to discriminate between an active or an inactive TB. In the study from Montuori et al. (39), apical consolidations reached a high specificity (92.2%) but showed a lower sensitivity (45.1%). Similarly, cavitations showed good specificity (89.3%) (39), but sensitivity was very low, ranging from 4.0 to 30.0% in the various studies (37–40). The explanation for these results relies in the fact that ultrasound is able to detect only evident pathological conditions located in the chest wall or at the pleuro-pulmonary interface when not obscured by bone structures of the chest wall. A composite of several TUS findings may provide acceptable sensitivity and specificity, especially in areas without ready access to standard radiologic exams where TB is endemic (39, 40). However, more studies in patients with and without TB are required. Despite these limitations, in confirmed cases of pulmonary TB, TUS may be used in the follow-up of subpleural consolidations to assess response to therapy. In such context, TUS may configure a suitable and repeatable alternative to others imaging methods implying radiation exposure, resulting useful especially in children and pregnant women. In addition, US-guided transthoracic cutting biopsy may represent a safe and minimally invasive technique for histological and microbiological characterization of TUS-accessible pulmonary consolidations, allowing to rule out other differential diagnosis.

Thoracic ultrasound (TUS) demonstrated good sensitivity in the detection of pleural effusion. A validated context for TUS employment is the qualitative study of pleural effusion with the detection of septated fluid collection. At the same time, TUS represents a useful real-time and risk-free guide for thoracentesis, drainage of loculated effusions and transthoracic biopsy of pleural plaques. In countries with good economic availability, diagnosis can further avail of TB cultures and a nucleic acid amplification (NAA) test. In areas with high tuberculosis prevalence, the diagnosis of pleural TB may be more simply suggested by the finding of lymphocytosis in a pleural effusion showing a complex septated pattern at TUS.

Given the advantageous cost-containment of this imaging technology, other useful applications of TUS in low- and middle-income settings are the detection of pericardial effusions and pericardiocentesis, which may be especially indicated in patients with HIV-associated TB and children. Last, but not least, TUS may serve as a safe and radiation-free method for guided needle aspiration/drainage of abscesses involving the anterior chest wall and paraspinal collections.

The main limitation of this summary report assessing the role of TUS in pulmonary TB is that, in the absence of controlled studies on large series of TB patients, even TUS usefulness in guiding interventional maneuvers can only be supported by small case series, single case reports and the clinical experience of the authors. Further research is to be encouraged to prove TUS beneficial, especially in countries where TB is endemic.

## Data Availability Statement

The original contributions presented in the study are included in the article/supplementary material, further inquiries can be directed to the corresponding author.

## Ethics Statement

Due to the descriptive nature of this manuscript, a written informed consent to participate in a clinical study was not required. The explanatory pictures included in this manuscript are from examinations performed as part of our routine medical practice for which patients signed informed consent. Patients provided informed permission for the images publication. The images have been anonymized to protect patients' privacy.

## Author Contributions

GR, MS, and DL contributed to the initial conception ad design of the work. All the authors contributed to draft the manuscript or revise it critically for important intellectual content and read and approved the submitted version.

## Conflict of Interest

The authors declare that the research was conducted in the absence of any commercial or financial relationships that could be construed as a potential conflict of interest.

## Publisher's Note

All claims expressed in this article are solely those of the authors and do not necessarily represent those of their affiliated organizations, or those of the publisher, the editors and the reviewers. Any product that may be evaluated in this article, or claim that may be made by its manufacturer, is not guaranteed or endorsed by the publisher.
